# Cucurbitacin E Induces Autophagy via Downregulating mTORC1 Signaling and Upregulating AMPK Activity

**DOI:** 10.1371/journal.pone.0124355

**Published:** 2015-05-13

**Authors:** Qing-Bing Zha, Xiao-Yu Zhang, Qiu-Ru Lin, Li-Hui Xu, Gao-Xiang Zhao, Hao Pan, Dan Zhou, Dong-Yun Ouyang, Ze-Huan Liu, Xian-Hui He

**Affiliations:** 1 College of Life Science and Technology, Jinan University, Guangzhou, China; 2 Department of Fetal Medicine, the First Affiliated Hospital of Jinan University, Guangzhou, China; Wayne State University School of Medicine, UNITED STATES

## Abstract

Cucurbitacins, the natural triterpenoids possessing many biological activities, have been reported to suppress the mTORC1/p70S6K pathway and to induce autophagy. However, the correlation between such activities is largely unknown. In this study, we addressed this issue in human cancer cells in response to cucurbitacin E (CuE) treatment. Our results showed that CuE induced autophagy as evidenced by the formation of LC3-II and colocalization of punctate LC3 with the lysosomal marker LAMP2 in HeLa and MCF7 cells. However, CuE induced much lower levels of autophagy in ATG5-knocked down cells and failed to induce autophagy in DU145 cells lacking functional ATG5 expression, suggesting the dependence of CuE-induced autophagy on ATG5. Consistent with autophagy induction, mTORC1 activity (as reflected by p70S6K and ULK1_S758_ phosphorylation) was inhibited by CuE treatment. The suppression of mTORC1 activity was further confirmed by reduced recruitment of mTOR to the lysosome, which is the activation site of mTORC1. In contrast, CuE rapidly activated AMPK leading to increased phosphorylation of its substrates. AMPK activation contributed to CuE-induced suppression of mTORC1/p70S6K signaling and autophagy induction, since AMPK knockdown diminished these effects. Collectively, our data suggested that CuE induced autophagy in human cancer cells at least partly via downregulation of mTORC1 signaling and upregulation of AMPK activity.

## Introduction

Cucurbitacins belong to a large family of triterpenoids present in Cucurbitaceae plants, and possess many biological activities, including anticancer, anti-inflammatory, anti-diabetic, and hepatoprotective activities [1]. The potential action targets of these triterpenoids or the molecular mechanism(s) underlying their activities have been investigated for decades. Some studies point to the inhibition of Jak/signal transducer and activator of transcription 3 (STAT3) signaling being the mechanism responsible for inhibitory effects of cucurbitacins on cancer cells [2], while others support the notion that it is the rapid disruption of the actin cytoskeleton by cucurbitacins being responsible for their anticancer activities [3, 4]. Cucurbitacins can disrupt the actin cytoskeleton rapidly, leading to a marked cell deformation, accompanied by the activation of several stress-sensing signaling pathways [3, 5]. Recent work indicated that cucurbitacins may target cofilin [6, 7], a critical regulator of actin dynamics, thereby damaging the actin cytoskeleton. Interestingly, cofilin-actin rods were formed in cells treated with cucurbitacin B and such rod formation relies on the over-activation of cofilin [8]. Therefore, multiple targets may be involved in the action of cucurbitacins.

Aside from their action on the actin cytoskeleton and Jak/STAT3 signaling, cucurbitacins have recently been shown to inhibit the mTORC1 activity in cancer cells [9]. Importantly, we and others found that cucurbitacins could induce a robust macroautophagy (hereafter referred to as autophagy) response in many cell types. We found that cucurbitacin B induced autophagy in Jurkat cells, which seemed to be a pro-survival response [10]. Likewise, Zhang *et al*. reported that cucurbitacin B and I induced autophagy by the production of mitochondrial-derived reactive oxygen species (ROS) through a STAT3-independent process [11]. However, our previous study showed that cucurbitacin B treatment did not elevate, but rather slightly reduced cellular ROS levels in human A375 and murine B16F10 cells [8]. Thus, other potential mechanism(s) may also contribute to cucurbitacin-induced autophagy. Given the critical role of mTORC1 in regulating autophagy [12–14], it will be of interest to determine the relationship between autophagy induction and suppression of the mTORC1 activity by cucurbitacins.

Autophagy is a homeostasis process in eukaryotes by which damaged organelles or proteins are sequestered inside double-membrane vesicles and delivered to the lysosome for degradation to recycle nutrients and energy upon exposure to various cellular stresses including drugs [15]. This process is critically regulated by the mTOR signaling. By interacting with other partners, the mTOR kinase forms two functionally distinct complexes, named mTOR complex 1 (mTORC1) and mTORC2 [16]. mTORC1 is a central regulator of autophagy [12–14], inhibition of which by its allosteric inhibitor rapamycin induces autophagy [17]. Many upstream signaling molecules, including AMP-activated protein kinase (AMPK), Erk1/2, tuberous sclerosis protein 2 (TSC2), and phosphatase and tensin homolog (PTEN), converge on mTORC1 to regulate autophagy [16, 18]. AMPK is an energy sensor that acts together with mTORC1 to maintain cellular homeostasis via modulating both the anabolic and catabolic processes [19]. Particularly, AMPK and mTORC1 regulate autophagy through coordinated phosphorylation of ULK1 (Unc51-like kinase, hATG1) at specific sites, respectively. AMPK promotes autophagy by phosphorylating (activating) ULK1 at Ser 317, 555 and 777 [13, 20], and by inhibiting mTORC1 at the level of TSC2 and Raptor (a substrate-recruiting subunit of mTORC1) [21, 22]. Conversely, high mTORC1 activity prevents ULK1 activation and attenuates autophagy by phosphorylating ULK1 Ser757 (human Ser758) and disrupting the interaction between ULK1 and AMPK [13].

As a member of cucurbitacin family and a close analog of cucurbitacin B, cucurbitacin E (CuE) exhibits similar biological activities to other cucurbitacins [23–25]. However, whether CuE induces autophagy and inhibits mTORC1 activity as well as the relationship between these processes is unknown. We found in this study that CuE induced autophagy at least partly via downregulation of mTORC1 signaling and upregulation of AMPK activity.

## Methods

### Materials

Cucurbitacin E was obtained from Shanghai Shunbo Chempharm Co (Shanghai, China). Chloroquine (CQ), dimethylsulfoxide (DMSO) and sodium dodecyl sulfate (SDS), polyformaldehyde were from Sigma-Aldrich (St. Louis, MO, USA). WST-1 was from Roche (Penzberg, Germany). Lipofectamine RNAiMAX, Dulbecco's modified Eagle's medium (DMEM), penicillin, streptomycin, L-glutamine, and fetal bovine serum (FBS) were from Gibco/ Invitrogen (Carlsbad, CA, USA). Polyvinylidene difluoride (PVDF) membranes (Hybond-P) were obtained from GE Healthcare Life Sciences (Piscataway, NJ, USA). Antibodies against p-p70S6K_T389_, p70S6K, p-4E-BP1_T37/46_, 4E-BP1, p-S6_S235/236_, S6, p62/SQSTM1, LC3B, ATG5, Beclin 1, p-AKT_S473_, p-Akt_T308_, Akt, p-AMPKα_T172_, AMPKα, p-AMPKβ1_S108_, AMPKβ1, p-ULK1_S758_, p-ULK1_S555_, ULK1, p-Raptor_S792_, Raptor, β-tubulin and HRP-conjugated second antibody were obtained from Cell Signaling Technology (Danvers, MA, USA). The antibody against LAMP2 was obtained from Abcam (Cambridge, MA, USA).

### Cell culture

HeLa, MCF7 and DU145 cells were obtained from the Cell Bank of the Chinese Academy of Sciences (Shanghai, China), and maintained in DMEM supplemented with 10% FBS, 100 U/ml penicillin and 100 μg/ml streptomycin at 37°C in a humidified incubator with 5% CO_2_.

### Cell viability assay

Cells in log-phase were seeded in 96-well plates (3300 cells/well) overnight and were then treated with indicated concentrations of CuE. Cell viability was assayed using WST-1 kit according to the procedures recommended by the supplier.

### Western blot analysis

Western blotting was performed as described previously [26]. The bands on PVDF membranes were revealed with an enhanced chemiluminescence kit (BeyoECL Plus; Beyotime, Haimen, China) and recorded on X-ray films (Kodak, Xiamen, China). The densitometry of each band was quantified by FluorChem 8000 (AlphaInnotech, San Leandro, CA, USA).

### Immunofluorescence microscopy

Immunofluorescence was performed essentially as previously reported [27, 28]. Briefly, cells were incubated with appropriate primary antibodies followed by incubation with CF488-conjugated goat-anti-mouse IgG and CF568-conjugated goat-anti-rabbit IgG (Biotium, Hayward, CA, USA). Fluorescence images were collected under a fluorescent microscope armed with a Spinning Disk Confocal Microscopy system (Ultra View cooled CCD; Perkin Elmer, Waltham, MA, USA).

### Small interfering RNA (siRNA)

siRNA knockdown was performed as described previously [26]. Briefly, cells were seeded in 6-well plates for 24 h followed by transfection with *ATG5* (Cell Signaling Technology) or *AMPKα* siRNA targeting 5′ACACATGAATGCAAAGATA3′ and 5′CCAGAAAGCTCTTCATAAA3′ (RiboBio, Guangzhou, China) for 72 h, respectively. Cells were then treated with indicated doses of CuE and collected for western blot analysis.

### Statistical analysis

All experiments were performed in triplicate, with one representative experiment shown. Data were expressed as mean ± SD. Statistical analysis was performed using GraphPad Prism 4.0 (GraphPad Software Inc., San Diego, CA). One-way ANOVA, followed by Dunnett’s multiple comparison tests (versus control), was used to analyze the statistical significance among multiple groups. *P* values < 0.05 were considered statistically significant.

## Results

### CuE-induced autophagy is dependent on ATG5 expression

**Fig 1:**
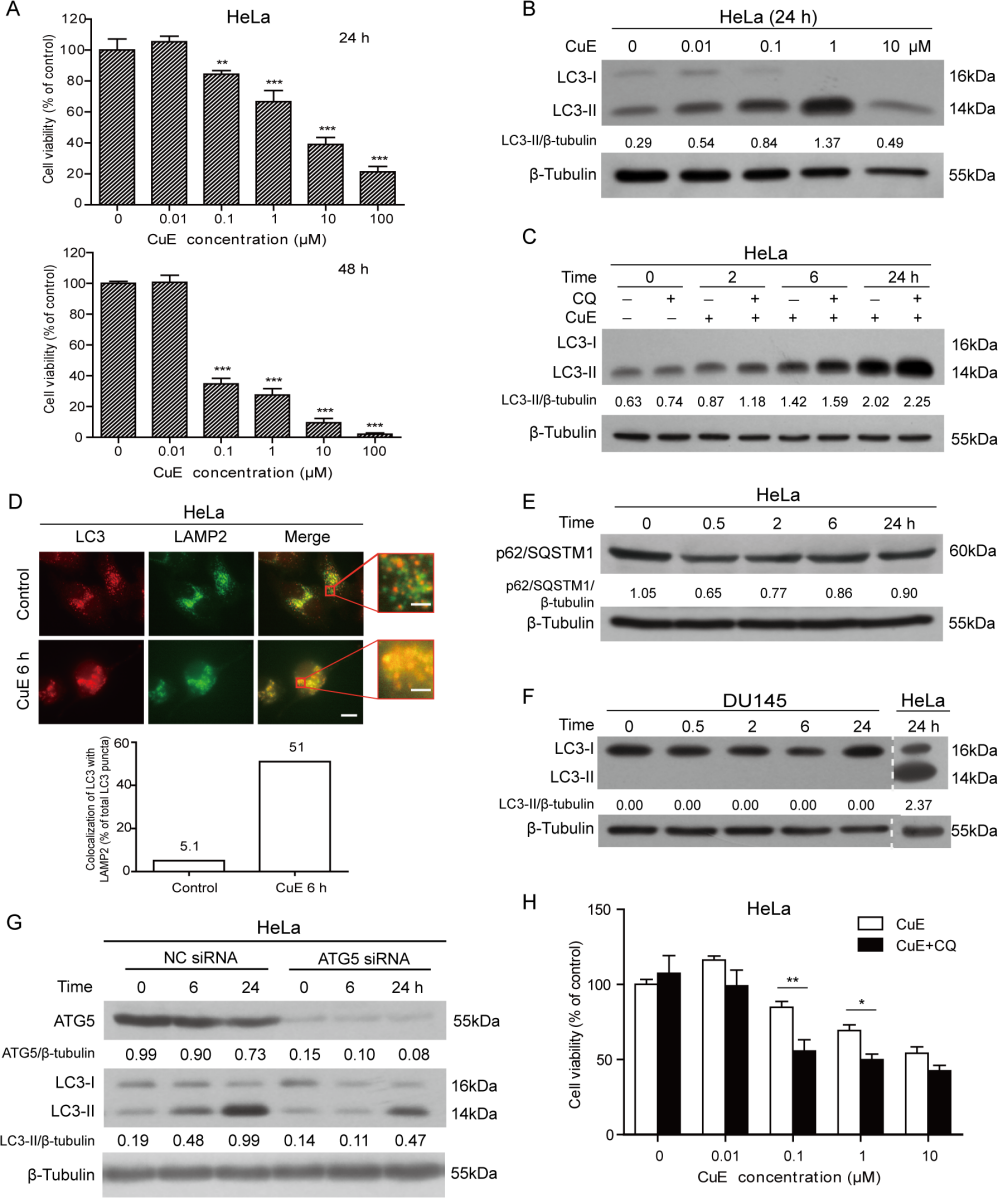
Autophagy induction by cucurbitacin E (CuE). (**A**) Cell proliferation was measured by WST-1 assay. (**B, C**) Western blot analysis of LC3 levels in HeLa cells treated with CuE for 24 h in the absence or presence of CQ. (**D**) Immunofluorescence microscopy showing the co-localization of LC3 and LAMP2. HeLa cells were cultured with CuE (1 μM) and then immunostained and visualized by fluorescent microscopy. Quantitative analysis is shown in the bottom panel. Scale bars: 10 μm (2 μm in magnified images). (**E**) Western blotting showing p62/SQSTM1 levels in HeLa cells treated with CuE (1 μM). (**F, G**) Western blot analysis of LC3 levels in DU145 cells (**F**), or in ATG5-knocked down HeLa cells (**G**), both of which were treated with CuE (1 μM). (**H**) HeLa cells were incubated with CuE in the presence of CQ (20 μM) for 24 h. Cell proliferation was measured by WST-1 assay. Values are shown as mean ± SD (n = 3). Representative blots of three independent experiments are presented and the relative densitometry ratios are shown under each band. β-Tubulin was used as a loading control. All blots are cropped to show only the target bands for clarity. *P <0.05, **P < 0.01, ***P < 0.001. NC, negative control.

We initially used a modified MTT (WST-1) assay to show that CuE dose-dependently inhibited the proliferation of HeLa cells (Fig 1A). The IC_50_ values were 4.01 μM and 0.06 μM for 24 h and 48 h, respectively. Based on these data, three CuE concentrations (0.1, 1 and 10 μM) were used for the following experiments with 1 μM concentration being used for most mechanistic assays. These same doses were used for the other two cell lines (MCF7 and DU145) as CuE had similar effect on them (S1A and S1B Fig).

Previous studies have revealed that cucurbitacins, including cucurbitacin B, IIa and I, can induce autophagy in several cell lines [10, 11, 29, 30]. As one of the most abundant member of the cucurbitacin family, it is still unknown whether CuE is capable of inducing autophagy in human cancer cells. Thus, we investigated this issue in HeLa and MCF7 cells by several assays that are commonly used to reveal autophagy induction [31]. First, the formation of LC3B-II was detected by western blotting. Upon autophagy induction, the diffusive cytoplasmic protein LC3 is conjugated with phosphatidylethanolamine (PE) on the pre-autophagosomal and autophagosomal membranes to yield punctate LC3-II, thus serving as a marker of autophagy. CuE induced accumulation of LC3-II in HeLa cells (Fig 1B) and MCF7 cells (S2A Fig). Second, the autophagic flux into the lysosomal compartment was also assayed by analyzing LC3-II formation in cells treated with CuE in the presence of chloroquine (CQ), an inhibitor of the lysosome. Western blotting showed that CuE plus CQ further increased the levels of LC3-II when compared with CQ alone (Fig 1C, S2B Fig), indicating an increased autophagic flux upon CuE treatment. Third, CuE-induced autophagy was confirmed by the colocalization of punctate LC3 with lysosomal-associated membrane protein 2 (LAMP2) using fluorescence microscopy. Under basal conditions, most of the punctate LC3 were separated from LAMP2 puncta; upon CuE treatment, however, most of the punctate LC3 were colocalized with LAMP2 (Fig 1D, S2C Fig), suggestive of increased fusion of autophagosomes with lysosomes. In addition, the level of p62/SQSTM1 has also been used for monitoring autophagy flux, since its levels are reduced upon autophagy induction [32, 33]; as an autophagy adaptor, p62/SQSTM1 binds directly to LC3-II and mediates the targeted degradation of ubiquitinated protein aggregates [34, 35]. Consistent with increased autophagy, p62/SQSTM1 levels were declined upon CuE treatment in HeLa (Fig 1E) and MCF7 cells (S2D Fig). Collectively, these results indicated that CuE induced an increase in autophagic flux in human cancer cells.

Since the DU145 cell line is defective in autophagy due to the lack of ATG5 [26], we used it to determine whether CuE-induced autophagy was dependent on ATG5 expression. As expected, CuE did not induce the formation of LC3-II in DU145 cells (Fig 1F). Neither LC3 punctum formation nor colocalization of LAMP2 with LC3 was observed in this cell line (S2F Fig). Consistent with this, ATG5 knockdown robustly diminished CuE-induced LC3-II formation in HeLa (Fig 1G) and MCF7 cells (S2E Fig). These data indicated that CuE-induced autophagy was dependent on ATG5 expression. We also detected the levels of beclin 1, a critical component for the initiation of autophagy, and found that it only slightly changed during CuE treatment (S2G Fig), in keeping with the concept that Beclin 1 is not recommended as an surrogate marker of autophagy [31]. Besides, when autophagy was blocked by CQ, CuE displayed increased cytotoxicity in HeLa cells (Fig 1H), suggesting that CuE-induced autophagy might be cytoprotective.

### CuE treatment downregulates both mTORC1/p70S6K and mTORC1/ULK1 signaling pathways

**Fig 2:**
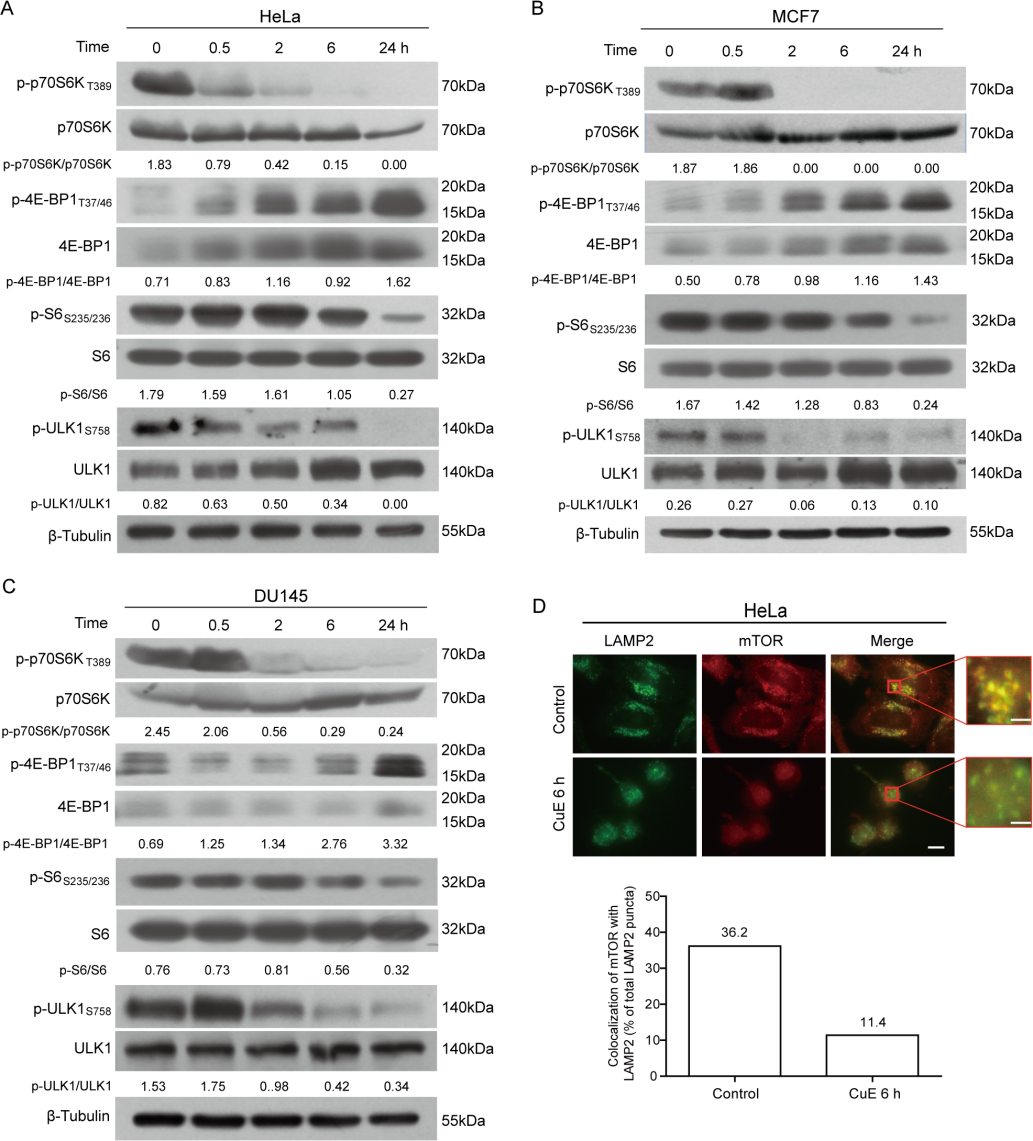
Effects of CuE on the mTORC1 signaling pathways. The phosphorylation of downstream signaling proteins of mTORC1 in HeLa (**A**), MCF7 (**B**) and DU145 (**C**) cells treated with CuE (1 μM) were assayed by western blotting. Representative blots of at least three independent experiments are presented and the relative densitometry ratios are shown under each band. All blots are cropped to show only the target bands for clarity. (**D**) Immunofluorescent analysis of mTOR colocalization with LAMP2. HeLa cells were cultured with CuE (1 μM) and then immunostained and visualized by fluorescent microscopy. Quantitative analysis is shown in the bottom panel. Scale bars: 10 μm (2 μm in magnified images).

Previous studies have reported that cucurbitacin B could suppress mTORC1/p70S6K signaling [9]. As mTORC1 signaling plays an important role in regulating autophagy under various stresses, we assayed whether CuE suppressed mTORC1 signaling by measuring the phosphorylation of p70S6K, S6 and 4E-BP1, the established markers of mTORC1 activity. Western blotting revealed that in HeLa, MCF7, and DU145 cells, CuE rapidly downregulated the levels of phosphorylated p70S6K_T389_ (Fig 2A–2C), indicative of decreased mTORC1 activity. Consistent with this, the phosphorylation levels of S6, a downstream substrate of p70S6K, was also decreased (Fig 2A–2C). In contrast, the phosphorylation of another mTORC1 substrate 4E-BP1_T37/46_ was not decreased but instead increased over time (Fig 2A–2C); 4E-BP1 expression was also increased during CuE treatment, but the underlying mechanism needs further investigation. As a direct substrate of mTORC1 and the autophagy-initiating kinase, the phosphorylation of ULK1_S758_ in all tested cells was suppressed in a time-dependent manner upon CuE treatment (Fig 2A–2C). To further confirm CuE-induced downregulation of mTORC1 activity, we detected the colocalization of mTOR with the lysosomal marker LAMP2, as the lysosomal surface is the site for mTORC1 activation by a complex process via the interaction with Rag guanosine triphosphatases and/or Rheb in response to many cues, including amino acids [36–38]. Under basal conditions, mTOR showed a high colocalization with LAMP2 puncta; but upon CuE treatment, mTOR had reduced colocalization with LAMP2 (Fig 2D), reflecting decreased mTORC1 activity [37]. Together, these results indicated that CuE suppressed both mTORC1/p70S6K and mTORC1/ULK1 signaling branches but not mTORC1/4E-BP1 signaling. Given the important role of mTORC1 in regulating autophagy, our data suggested that suppression of mTORC1 activity by CuE might contribute to its autophagy-inducing effect.

### CuE activates AMPK signaling but not Akt signaling upstream to mTORC1

**Fig 3:**
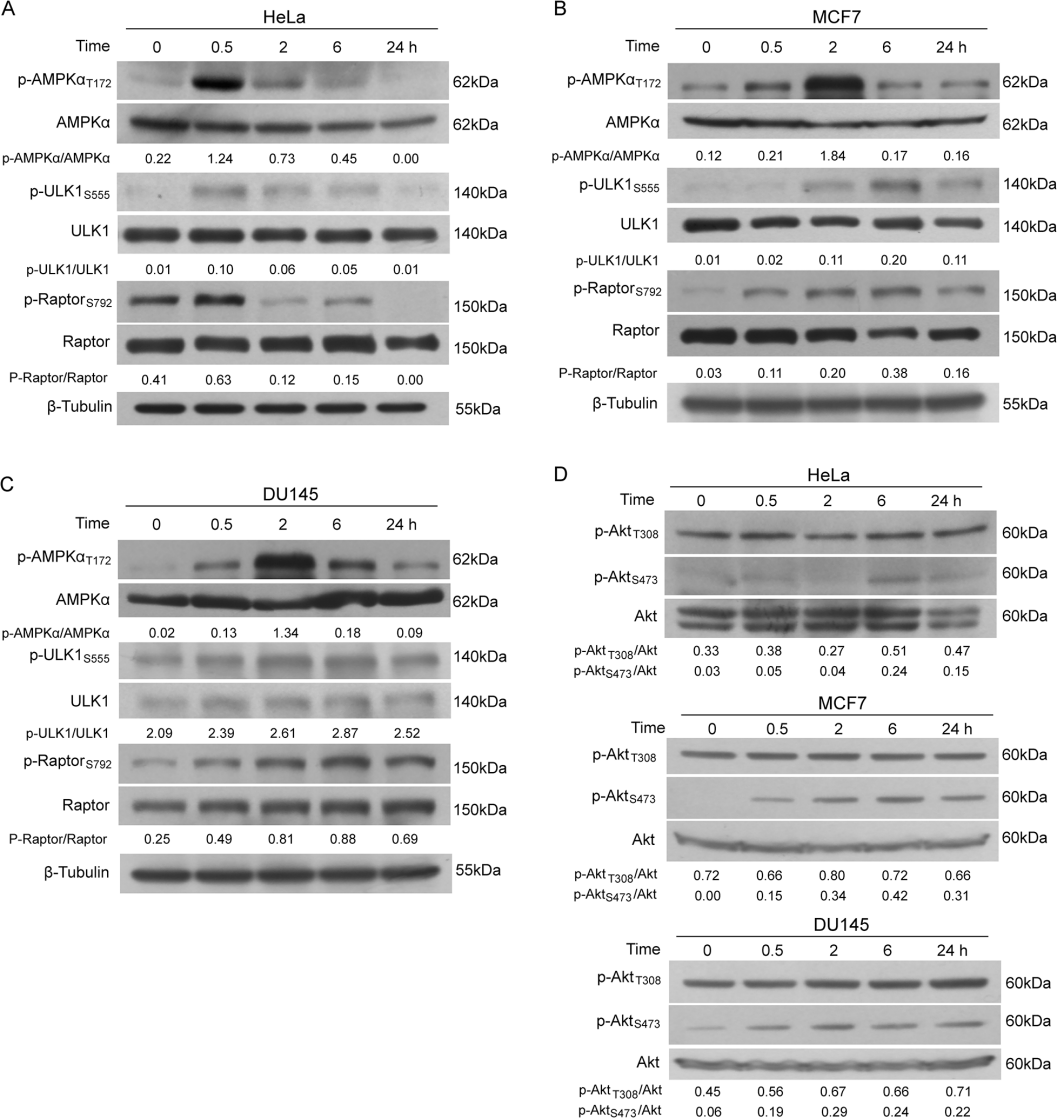
Effects of CuE on the upstream signaling of mTORC1. HeLa (**A, D**), MCF7 (**B, D**) and DU145 (**C, D**) cells were cultured with CuE (1 μM) and subjected to western blot analysis. Representative blots of three independent experiments are presented and the relative densitometry ratios are shown under each band. All blots are cropped to show only the target bands for clarity.

As mTORC1 activity is regulated by upstream kinases including AMPK and Akt [16], we next investigated whether these upstream regulators were involved in CuE-induced suppression of mTORC1 activity. We found that, upon CuE treatment, the phosphorylation (activation) levels of AMPKα was increased robustly at early time points but gradually returned back to basal levels at later time points (Fig 3A–3C). Similar changes in the phosphorylation levels of AMPKβ1 subunit was observed in CuE-treated cells (S3A–S3C Fig). Consistently, the AMPK substrates ULK1_S555_ and Raptor_S792_ showed correspondingly changes in its phosphorylation levels (Fig 3A–3C). As Raptor is the substrate-recruiting subunit of mTORC1, its phosphorylation at S792 by AMPK suggested that CuE-induced AMPK activation might contribute to the inhibitory effect of CuE on mTORC1 activity. On the other hand, Akt_T308_ phosphorylation was not influenced by CuE treatment (Fig 3D), suggesting that the upstream Akt signaling may not participate in this process. However, Akt_S473_ phosphorylation downstream of mTORC2 was slightly enhanced (Fig 3D), suggesting a loss of negative feedback of p70S6K signaling to the mTORC2 activity.

### Blocking AMPK activity attenuates CuE-induced mTORC1/p70S6K downregulation and autophagy

**Fig 4:**
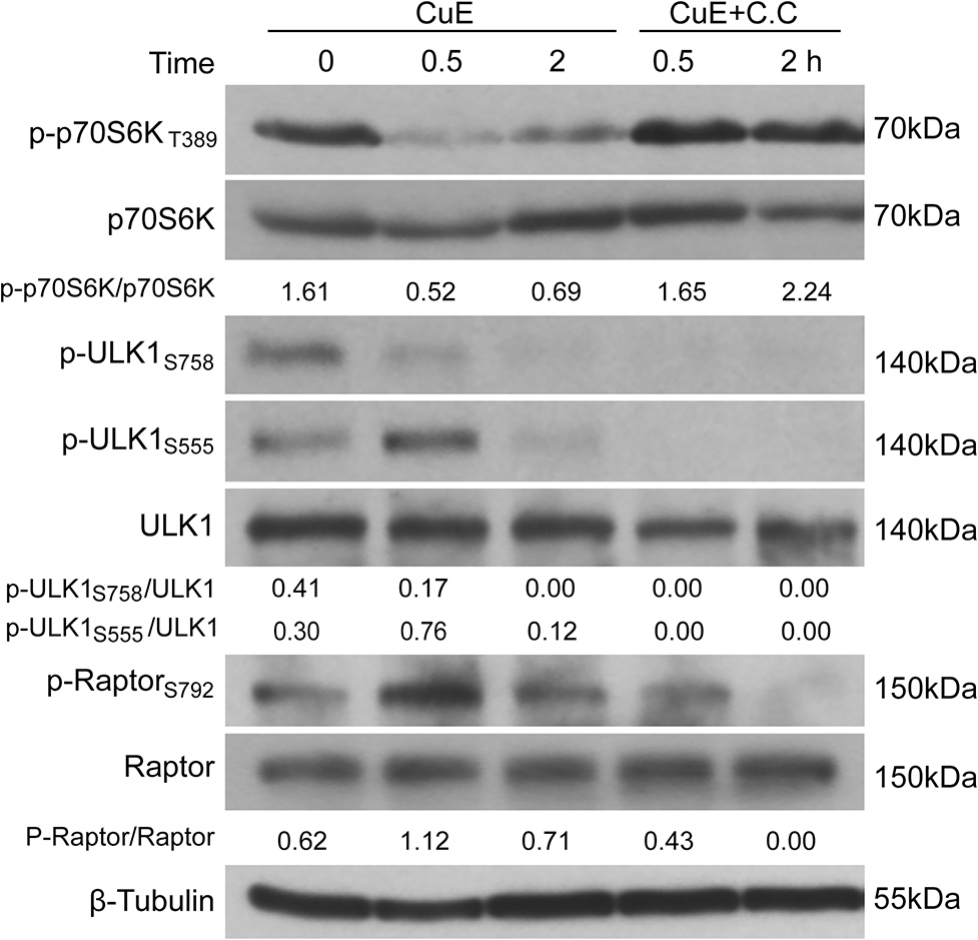
Effect of AMPK inhibitor on CuE-induced downregulation of mTORC1 signaling. HeLa cells were treated with CuE (1 μM) in the presence or absence of compound C (C.C) (20 μM) and subjected to western blotting. Representative blots of three independent experiments are presented and the relative densitometry ratios are shown under each band. All blots are cropped to show only the target bands for clarity.

As the aforementioned data suggested that CuE-induced activation of AMPK contributed to the inhibition of mTORC1 activity, we next explored the relationship between AMPK and mTORC1 by using an AMPK-specific inhibitor compound C (C.C) [39]. As expected, the phosphorylation of AMPK substrates ULK1_S555_ and Raptor_S792_ was suppressed below basal levels or even undetectable when treated with CuE in the presence of C.C in HeLa cells (Fig 4). Interestingly, CuE-induced suppression of p70S6K phosphorylation was completely reversed in HeLa cells and partially in MCF7 cells upon C.C treatment (Fig 4, S4 Fig), suggesting that the activation of AMPK contributed to the inhibition of mTORC1/p70S6K signaling. However, C.C was unable to restore the decreased phosphorylation levels of ULK1_S758_ caused by CuE treatment. These results suggested that the activation of AMPK was involved in the suppression of the mTORC1/p70S6K signaling but not the mTORC1/ULK1_S758_ signaling branch.

**Fig 5:**
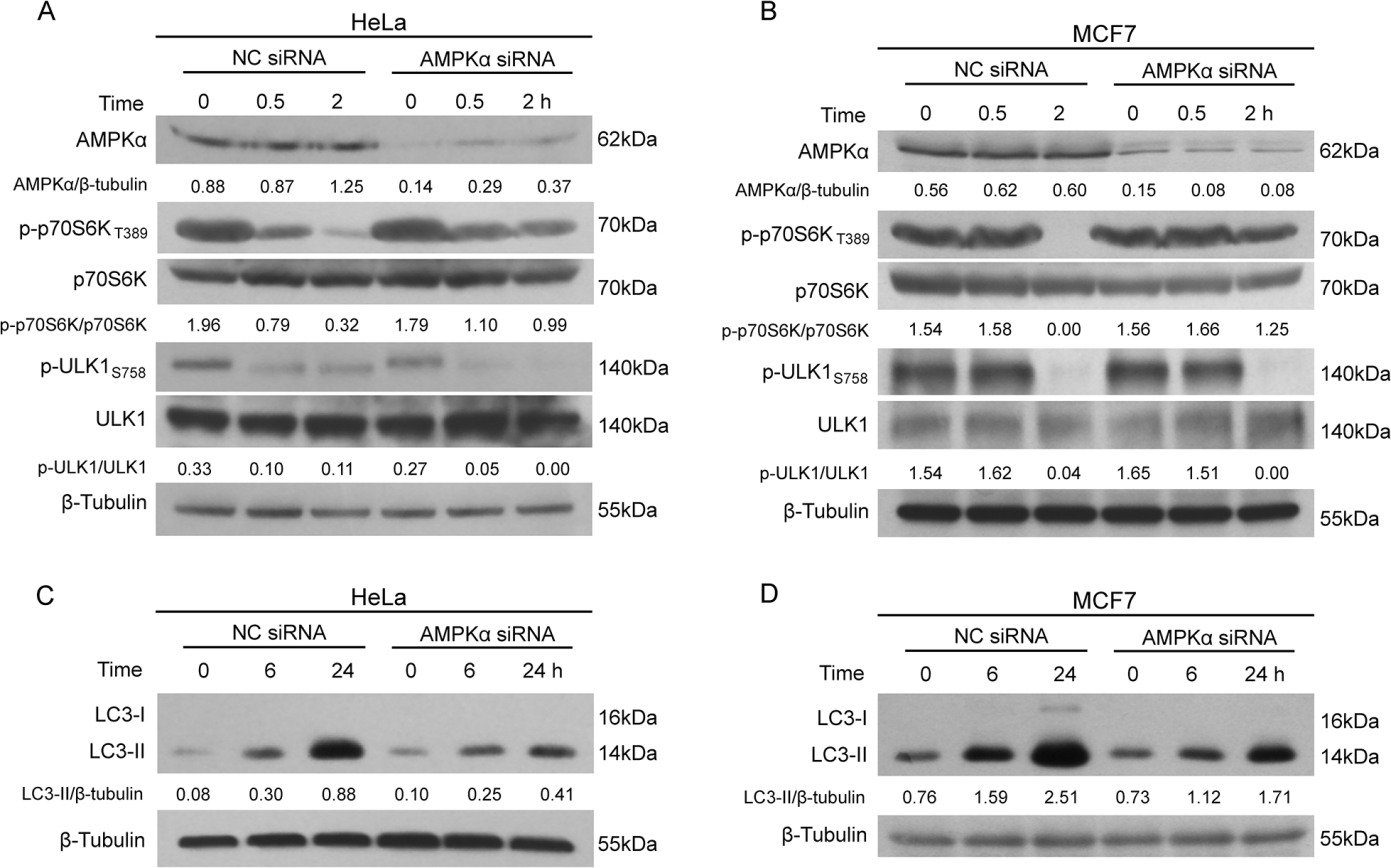
Effect of AMPK knockdown on CuE-induced downregulation of mTORC1 signaling and autophagy. HeLa (**A, C**) and MCF7 (**B, D**) cells were transfected with AMPKα siRNA or negative control (NC) siRNA for 72 h, and then treated with CuE (1 μM) for indicated times. Cells were subjected to western blot analysis of the mTORC1 signaling pathways (**A, B**) and LC3 levels (**C, D**). Representative blots of three independent experiments are presented and the relative densitometry ratios are shown under each band. All blots are cropped to show only the target bands for clarity.

To confirm the pharmacological results, we knocked down AMPKα with siRNAs specific for AMPKα1 and α2 in HeLa and MCF7 cells. Consistent with the action of C.C, AMPKα knockdown counteracted CuE-induced suppression of the mTORC1/p70S6K signaling, but not the mTORC1/ULK1_S758_ signaling branch (Fig 5A and 5B); the relationship between AMPK and mTORC1/ULK1_S758_ remains to be elucidated. Importantly, CuE-induced autophagy was significantly attenuated by AMPKα knockdown in HeLa and MCF7 cells (Fig 5C and 5D), further confirming the AMPK’s role in CuE-induced autophagy.

## Discussion

The present study revealed that CuE suppressed mTORC1 activity as evidenced by the decreased phosphorylation of its direct substrates p70S6K and ULK1, concomitant with the induction of autophagy in human cancer cells. The decreased phosphorylation of p70S6K was reversed by inhibiting AMPK activity. Furthermore, CuE-induced autophagy was dependent on the AMPK activity and correlated with mTORC1/p70S6K signaling. These data suggested that the upregulation of AMPK activity and downregulation of mTORC1 signaling contributed to autophagy induction upon CuE treatment.

Similar to other members of cucurbitacins, CuE has been showed to induce autophagy in the present study, which is supported by several lines of evidence. In HeLa and MCF7 cells with normal ATG5 expression, LC3-II accumulation was induced by CuE treatment and such an effect was further enhanced with co-treatment of CQ. Further analysis revealed increased colocalization of LC3 puncta with the lysosome marker LAMP2, indicating an increased autophagic flux. In contrast, there was no LC3-II band and LC3 punctum formation in CuE-treated DU145 cells which is lack of functional ATG5 expression [26] or reduced LC3-II formation in ATG5-knocked down HeLa and MCF7 cells. Thus, CuE-induced autophagy was dependent on ATG5 expression, in agreement with the previous study showing that cucurbitacin-I-induced autophagy requires ATG5 and Beclin 1 expression [11, 30].

Our finding that CuE-induced autophagy was concomitant with the suppression of mTORC1 activity identifies a potential link between these two processes. mTORC1 is a crucial regulator of autophagy by conveying a variety of environmental cues including energy status, nutrient availability, and growth factor signaling [16, 40]. Autophagy induction by inhibiting the mTORC1 activity has been widely used as a model or positive autophagy control [16]. Mechanically, high mTORC1 activity prevents autophagy by phosphorylating ULK1 at Ser758, thereby disrupting the interaction between ULK1 and AMPK [13]. When mTORC1 is inhibited, ULK1_S758_ is dephosphorylated, leading to the activation of ULK1 kinase. Meanwhile, this restores the interaction between ULK1 and AMPK, with AMPK further promoting ULK1 activity by directly activating ULK1 through phosphorylation of the other sites than Ser758 [13, 20]. Activated ULK1 phosphorylates ATG13 and FIP200, and ultimately induces autophagy [41]. We found in this study that CuE not only inhibited mTORC1/p70S6K signaling but also suppressed the mTORC1/ULK1 pathway. As mTORC1/p70S6K signaling is mainly involved in the regulation of protein translation [42]; the suppressed AMPK/ULK1 pathway was likely responsible for initiating CuE-induced autophagy. This notion was supported by the observation that AMPK knockdown markedly attenuated CuE-induced autophagy. Thus, both downregulation of the mTORC1 signaling and upregulation of AMPK activity were involved in CuE-induced autophagy.

As a member of cucurbitacins, CuE-induced suppression of mTORC1 activity, but not Akt, was consistent with previous observations performed on other cucurbitacins. It was reported that cucurbitacin B suppresses TNF-induced activation of mTORC1/p70S6K signaling although such effects was not significant under basal conditions [43]. While we were preparing this manuscript, a paper was published, showing that cucurbitacin I also inhibits mTORC1/p70S6K activity while activating AMPK but not Akt signaling in Glioblastoma cells [30]. Thus, suppression of the mTORC1/p70S6K signaling pathway seems a common property for cucurbitacins including cucurbitacins B, E and I.

Moreover, we further characterized the distinct sensitivity of mTORC1/p70S6K and mTORC1/ULK1_S758_ signaling branches to AMPK activity induced by CuE treatment, thus identifying a potential AMPK-independent suppression of mTORC1 activity. Although the increased phosphorylation of Akt_S473_ could be explained by a loss of the negative feedback loop by which p70S6K-mediated phosphorylation of Rictor inhibits mTORC2 and Akt signaling [44], it is still unknown why mTORC1/p70S6K_T389_ is sensitive to AMPK but mTORC1/ULK1_S758_ is not. One possible explanation is that there exist specific subsets of mTORC1 phosphorylation sites as was shown by a study demonstrating that distinct mTORC1 phosphorylation sites are affected differently by rapamycin [45]. For example, rapamycin inhibits the mTORC1 activity on the phosphorylation of p70S6K_T389_ but not 4E-BP1_T37/46_. Consistent with rapamycin, CuE suppressed p70S6K_T389_ but not 4E-BP1_T37/46_ phosphorylation. Interestingly, the phosphorylation site of ULK1_S578_ is also an established rapamycin-insensitive site [45]. However, phosphorylation of ULK1_S758_ was shown to be suppressed by CuE and such an effect was independent of AMPK activity. Hence, the notion that mTORC1 phosphorylation sites determine their sensitivity to rapamycin seems unable to resolve this phenomenon. Additional mechanism underlying this awaits further investigation.

In conclusion, we found in this study that, concomitant with the induction of autophagy, CuE was able to suppress the mTORC1 activity leading to decreased phosphorylation of its downstream substrates. CuE-induced autophagy seems to be mediated via modulating both the mTORC1 signaling pathway and the AMPK activity. Although it is unclear how CuE acts on AMPK or mTORC1 (directly or indirectly), our findings provided a basis for further research on the action mechanism(s) by which cucurbitacins (including CuE) act as anti-inflammatory, anti-diabetic, and anticancer agents.

## Supporting Information

S1 Fig Effect of cucurbitacin E (CuE) on the proliferation of MCF7 (A) and DU145 (B) cells. Cells were incubated with indicated doses of CuE for 24 h and 48 h, respectively. Cell viability was measured by WST-1 assay. Values are shown as mean ± SD (n = 3). *P <0.05, **P < 0.01 and ***P < 0.001 versus control (0 group).
(TIF)

S2 Fig Autophagy induction by cucurbitacin E (CuE). (**A**, **B**) Western blot analysis of LC3 levels in MCF7 cells treated with CuE (1 μM) for indicated time periods in the absence or presence of chloroquine (CQ). (**C**) Immunofluorescence microscopy showing the colocalization of LC3 and LAMP2 in MCF7 cells. Cells were cultured with CuE and then immunostained and visualized by fluorescent microscopy. Scale bars: 10 μm (2 μm in magnified images). (**D**) Western blotting showing p62/SQSTM1 levels in MCF7 cells treated with CuE. (**E**) Western blot analysis of ATG5 and LC3 levels in ATG5-knocked down MCF7 cells treated with CuE. (**F**) Immunofluorescence microscopy showing the distribution of LC3 and LAMP2 in DU145 cells. Scale bar: 10 μm (2 μm in magnified images). (**G**) Western blot analysis of Beclin 1 levels in HeLa, MCF7 and DU145 cells treated with CuE, respectively. The relative densitometry ratios are shown under each band. β-Tubulin was used as a loading control. NC, negative control.
(TIF)

S3 Fig Effect of CuE on AMPKβ phosphorylation. Cells were treated with CuE (1 μM) for indicated time periods and subjected to western blot analysis. The relative densitometry ratios are shown under each band. β-Tubulin was used as a loading control.
(TIF)

S4 Fig Effect of AMPK inhibitor on CuE-induced downregulation of p70S6K. HeLa (A), MCF7 (B) and DU145 (C) cells were treated with CuE (1 μM) in the presence or absence of compound C (C.C) (20 μM) and subjected to western blot analysis. The relative densitometry ratios are shown under each band. β-Tubulin was used as a loading control.
(TIF)
