# Supplementary figures and images for: Cucurbitacin E Induces Autophagy via Downregulating mTORC1 Signaling and Upregulating AMPK Activity
Source: PLoS One. 2015 May 13;10(5):e0124355. doi: 10.1371/journal.pone.0124355 (PMC4430304; doi:10.1371/journal.pone.0124355)

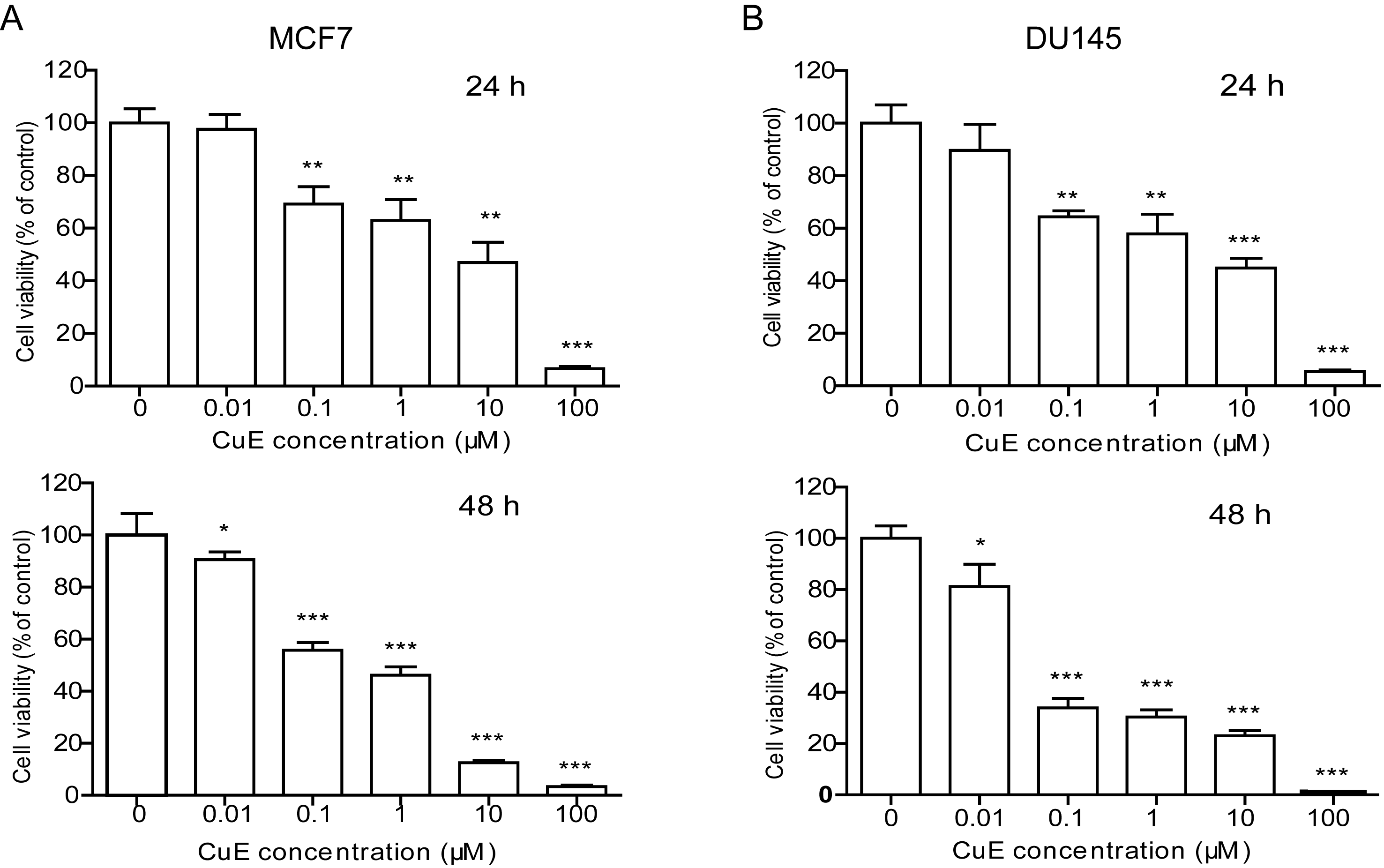

Supplement: S1 Fig — Cells were incubated with indicated doses of CuE for 24 h and 48 h, respectively. Cell viability was measured by WST-1 assay. Values are shown as mean ± SD (n = 3). *P <0.05, **P < 0.01 and ***P < 0.001 versus control (0 group). (TIF) [file pone.0124355.s001.tif]

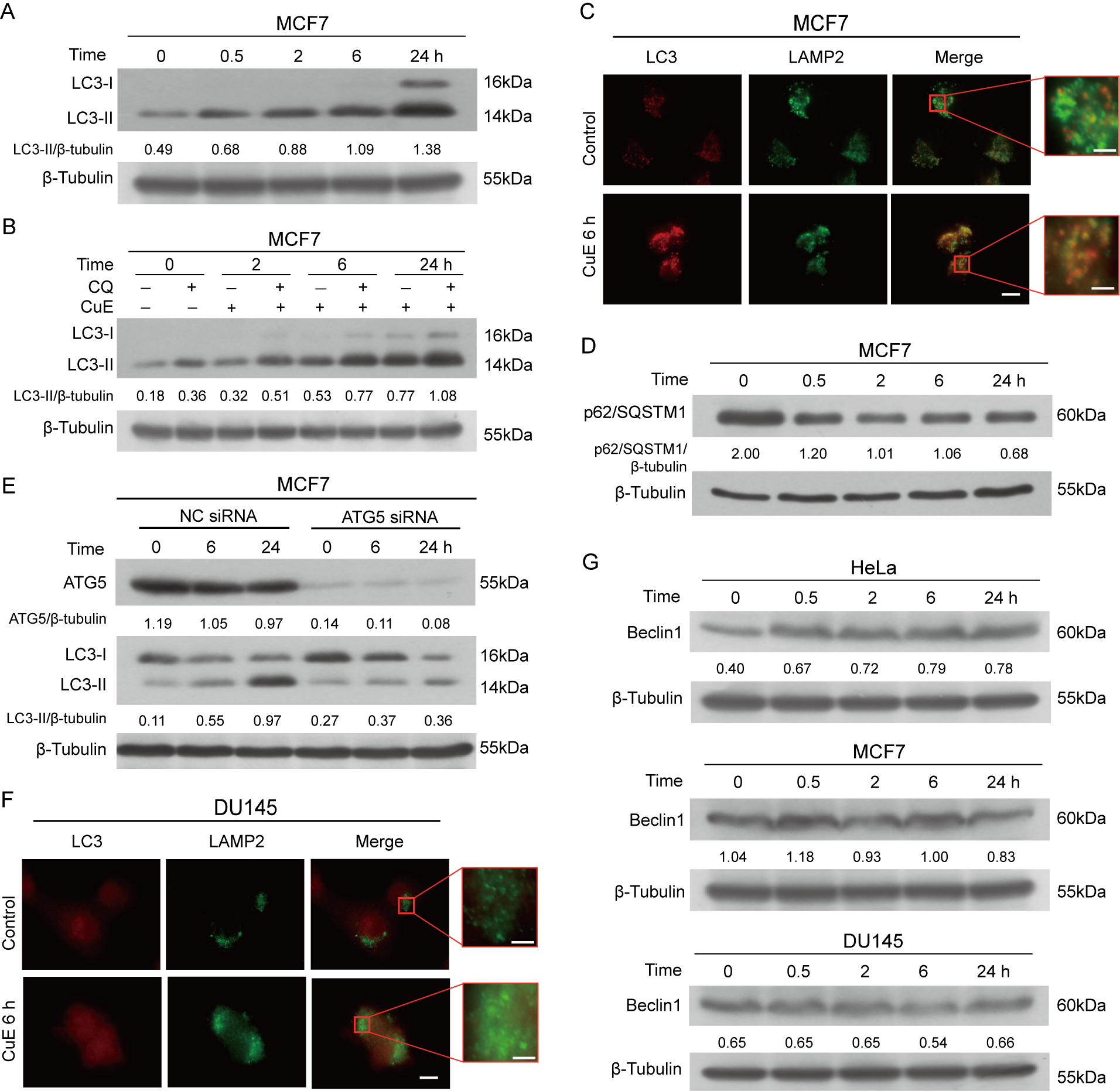

Supplement: S2 Fig — (A, B) Western blot analysis of LC3 levels in MCF7 cells treated with CuE (1 μM) for indicated time periods in the absence or presence of chloroquine (CQ). (C) Immunofluorescence microscopy showing the colocalization of LC3 and LAMP2 in MCF7 cells. Cells were cultured with CuE and then immunostained and visualized by fluorescent microscopy. Scale bars: 10 μm (2 μm in magnified images). (D) Western blotting showing p62/SQSTM1 levels in MCF7 cells treated with CuE. (E) Western blot analysis of ATG5 and LC3 levels in ATG5-knocked down MCF7 cells treated with CuE. (F) Immunofluorescence microscopy showing the distribution of LC3 and LAMP2 in DU145 cells. Scale bar: 10 μm (2 μm in magnified images). (G) Western blot analysis of Beclin 1 levels in HeLa, MCF7 and DU145 cells treated with CuE, respectively. The relative densitometry ratios are shown under each band. β-Tubulin was used as a loading control. NC, negative control. (TIF) [file pone.0124355.s002.tif]

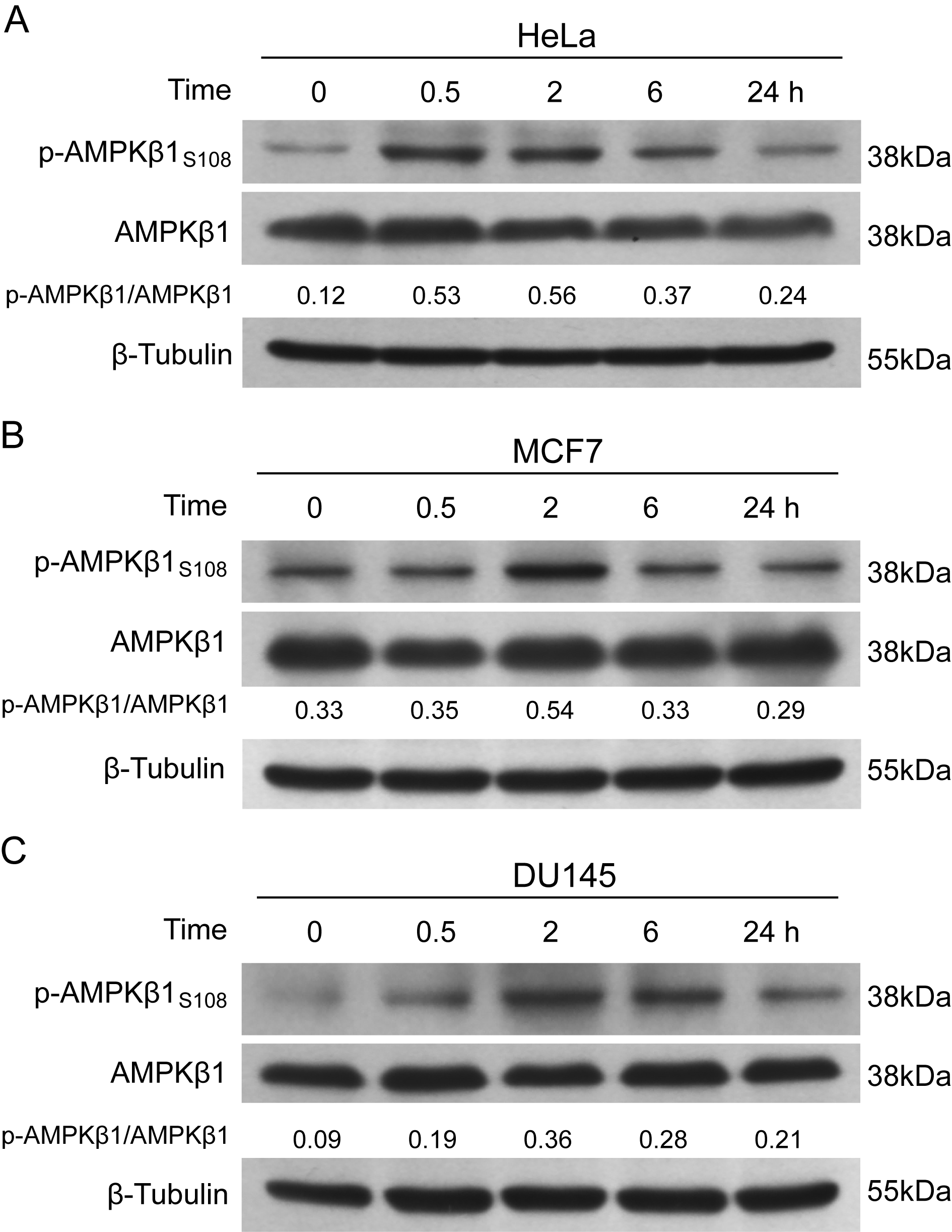

Supplement: S3 Fig — Cells were treated with CuE (1 μM) for indicated time periods and subjected to western blot analysis. The relative densitometry ratios are shown under each band. β-Tubulin was used as a loading control. (TIF) [file pone.0124355.s003.tif]

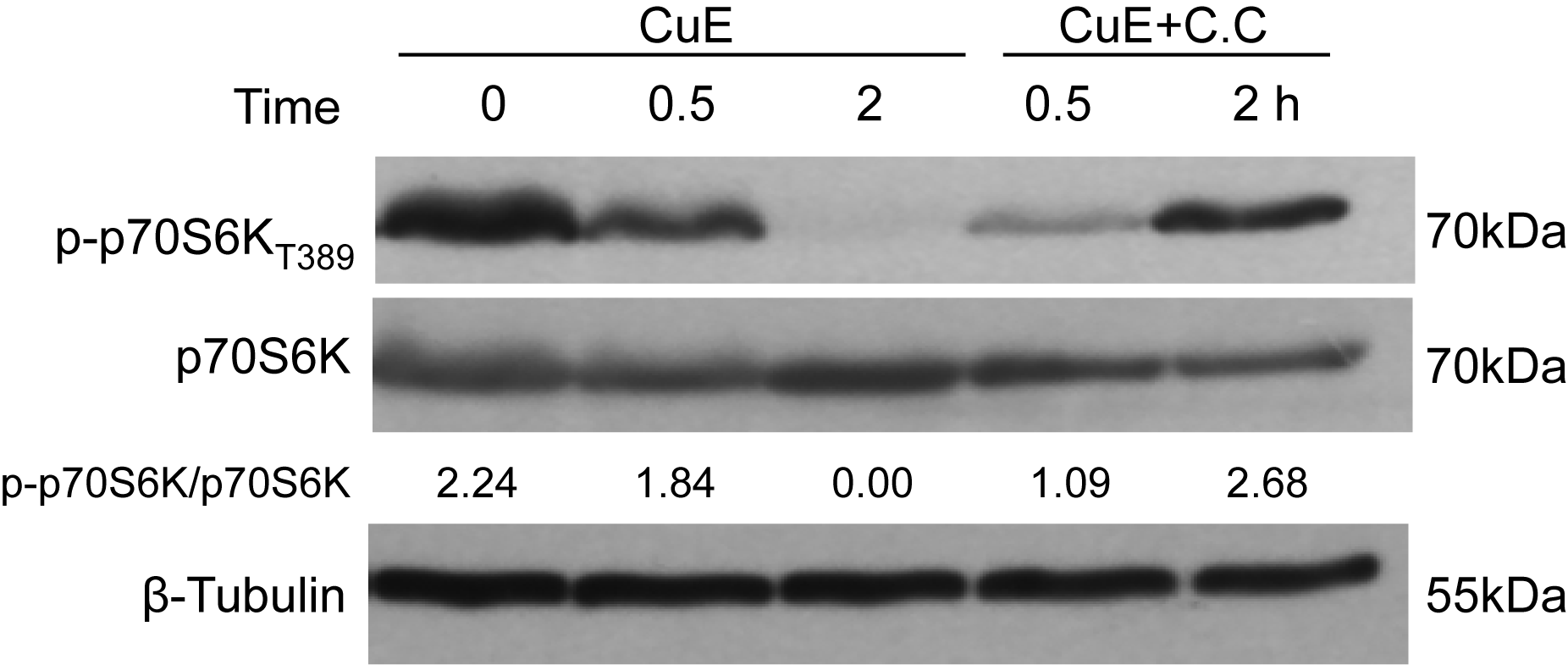

Supplement: S4 Fig — HeLa (A), MCF7 (B) and DU145 (C) cells were treated with CuE (1 μM) in the presence or absence of compound C (C.C) (20 μM) and subjected to western blot analysis. The relative densitometry ratios are shown under each band. β-Tubulin was used as a loading control. (TIF) [file pone.0124355.s004.tif]
